# Molecular and serological survey of *Trypanosoma vivax* in Crioulo Lageano Cattle from southern Brazil

**DOI:** 10.1590/S1984-29612025019

**Published:** 2025-04-14

**Authors:** Felipe Eduardo Fiorin, Mariana da Silva Casa, Leonardo Bergmann Griebeler, Mariana Fuchs Goedel, Gianlucca Simão Nadal Ribeiro, Luiz Flávio Nepomuceno do Nascimento, Gabriella Bassi das Neves, Graziela Vieira Fonteque, Luiz Cláudio Miletti, Mere Erika Saito, Joandes Henrique Fonteque

**Affiliations:** 1 Programa de Pós-graduação em Ciência Animal, Universidade do Estado de Santa Catarina – UDESC, Lages, SC, Brasil; 2 Hospital de Clínicas Veterinárias, Faculdade de Veterinária, Universidade Federal de Pelotas – UFPel, Pelotas, RS, Brasil; 3 Departamento de Medicina Veterinária, Universidade do Estado de Santa Catarina – UDESC, Lages, SC, Brasil; 4 Departamento de Medicina Veterinária, Universidade de Patos de Minas – UNIPAM, Patos de Minas, MG, Brasil; 5 Departamento de Medicina Veterinária, Centro Universitário FACVEST, Lages, SC, Brasil; 6 Departamento de Produção Animal e Alimentação, Universidade do Estado de Santa Catarina – UDESC, Lages, SC, Brasil

**Keywords:** Locally adapted breed, sanity, Trypanosomiasis, hematology, biochemistry, Raça localmente adaptada, sanidade, Tripanossomose, hematologia, bioquímica

## Abstract

Bovine trypanosomiasis, caused by infection with the protozoan *Trypanosoma vivax*, is harmful to livestock worldwide. Knowing its epidemiology is relevant to evaluate the susceptibility, resistance, and tolerance of animals. The objective of this study was to determine the prevalence of *T. vivax* in Crioula Lageana cattle and relate them to clinical, hematological, and biochemical findings to elucidate the breed’s health and disease tolerance characteristics. Venous blood samples from 310 bovines considered healthy during the clinical examination were used to perform polymerase chain reaction (PCR) analysis, Immunofluorescence Antibody Test (IFAT) assays, hemogram tests, and serum biochemistry. The collected data were subjected to statistical analyses to compare seropositive and negative groups. IFAT indicated that the seroprevalence for *T. vivax* was 8% (24/310); however, all tested animals were negative in the conventional PCR (0%, 0/310). Higher amounts of platelets and less cholesterol were detected in seropositive animals but were within the reference values. Ruminal hypomotility and mild tachycardia were observed in all sampled animals. Considering the non-specific clinical signs and the absence of hematological alterations in infected animals, the seropositivity found indicates previous exposure to the protozoan, and the absence of clinically affected animals may result from characteristics inherent to this breed.

## Resumo

A tripanossomíase bovina, causada pela infecção pelo protozoário*Trypanosoma vivax*, é prejudicial ao gado em todo o mundo. O conhecimento de sua epidemiologia é relevante para avaliar a suscetibilidade, resistência e tolerância desses animais. O objetivo deste estudo foi determinar a prevalência e soroprevalência de*T. vivax*em bovinos da raça Crioula Lageana e relacioná-las aos achados clínicos, hematológicos e bioquímicos, para elucidar as características de saúde e tolerância a doenças dessa raça. Amostras de sangue venoso de 310 bovinos, considerados saudáveis durante o exame clínico, foram utilizadas para realizar análises de reação em cadeia da polimerase (PCR), ensaios de imunofluorescência indireta (IFAT), hemogramas e bioquímica sérica. Os dados coletados foram submetidos a análises estatísticas para comparar os grupos soropositivos e soronegativos. A RIFI indicou que a soroprevalência do *T. vivax*foi de 8% (24/310); no entanto, todos os animais mostraram-se negativos na PCR convencional (0%, 0/310). Quantidades maiores de plaquetas e menos colesterol foram detectadas em animais soropositivos, mas estavam dentro dos valores de referência. Hipomotilidade ruminal e taquicardia leve foram observadas em todos os animais amostrados. Considerando os sinais clínicos inespecíficos e a ausência de alterações hematológicas e de animais infectados, a soroprevalência encontrada indica exposição prévia ao protozoário, e a ausência de animais clinicamente afetados pode resultar de características inerentes a essa raça.

## Introduction

Trypanosomiasis caused by *Trypanosoma vivax* occurs worldwide and is transmitted by blood-sucking dipteran flies, with tropical and subtropical regions being the most affected (Bastos et al., 2020), vertical transmission (Melo-Junior et al., 2024), and fomites (Leal et al., 2025) This disease causes significant economic impacts due to decreased productivity, treatment costs, and animal mortality (Bastos et al., 2015). Some genetic groups of cattle are resistant to trypanosomiasis. Using trypanotolerant breeds is a potent control and prevention tool that maintains productive capacity even after infection (Doko et al., 1991).

The Crioulo Lageano breed has excellent productive characteristics and an aptitude for producing meat (Mitterer-Daltoé et al., 2012; Mariante & Cavalcante, 2000). Epidemiological surveys anchored to molecular techniques associated with serological tests provide more accurate and reliable data regarding the real sanitary status of these herds (Fidelis et al., 2019).

Trypanosomiasis may commonly present as a subclinical infection in cattle, enabling them to be considered reservoirs (Sharma et al., 2013) or can manifest severe hematological alterations (Batista et al., 2008). Clinical disease outbreaks have been reported in populations with enzootic instability status for the agent. These populations are more prone to infection owing to their low acquired immunity, which is an important point to be questioned when considering locally adapted breeds with small populations of animals, such as the Crioulo Lageano breed (Smith et al., 2000).

Previous studies have suggested that *Bos indicus* lineages are more innately resistant to hemoparasite infection than *Bos taurus* (Jonsson, 2006). Cattle of the Crioulo Lageano breed, originating from *Bos taurus* lineages, are extremely rustic, adaptable, and tolerable to diseases and precarious conditions. There are *in situ* conservation centers for the maintenance of this population, which is an important source of genetic resources (Martins et al., 2009).

Knowledge of epidemiological data regarding the infection status of Crioulo Lageano breed, consisting of essential information to meet the requirements for commercialization of products of animal origin, is crucial for establishing the real sanity status of these herds as subclinical carriers are a source of infection for other animals (Ventura et al., 2001).

The aim of this study was to present the first survey of *T. vivax* in Crioulo Lageano cattle using molecular and serological techniques, as well as to compare physical examination variables, blood count, and serum biochemistry between seronegative and seropositive animals.

## Materials and Methods

### Sample size determination

The following formulas were used to assess the prevalence of *T. vivax* in the population of Crioulo Lageano cattle according to Thrusfield (2007):


$$n_{o}=\frac{1.96^{2}\left[p\left(1-p\right)\right]}{\left(d\right)²}$$ (1)

where *n_o_* is the number of samples, *p* is the expected prevalence, and *d* is the error margin. The obtained result was 384 animals, assuming an estimated prevalence of 50% positive samples, a 95% confidence interval, and a margin of error of 5%. However, the following calculation was performed because it consisted of a finite population:


$$n\;=\frac{N\;x\;n\text{o}}{N+\;n\text{o}}$$ (2)

where *N* is the total number of animals in the population (1,500 animals of the Crioulo Lageano breed). These calculations indicated that 306 animals should be sampled.

### Animals and collection of samples

**Figure 1:**
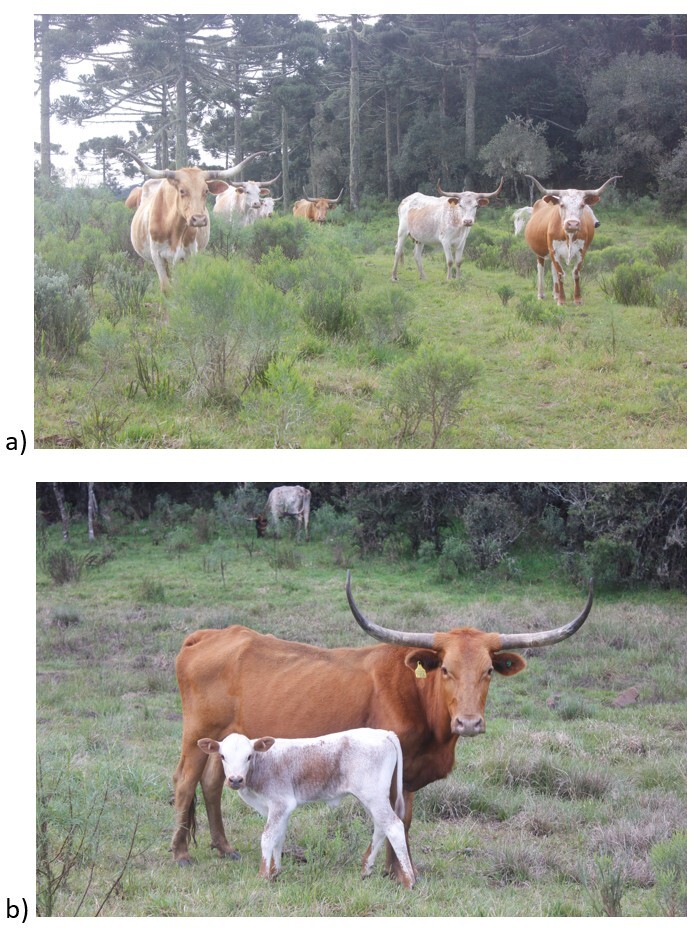
Crioula Lageana Cattle specimens (a,b), extensively reared on native pasture, from a *in situ* conservation nucleus, in the municipality of Painel, SC, Brazil.

**Figure 2:**
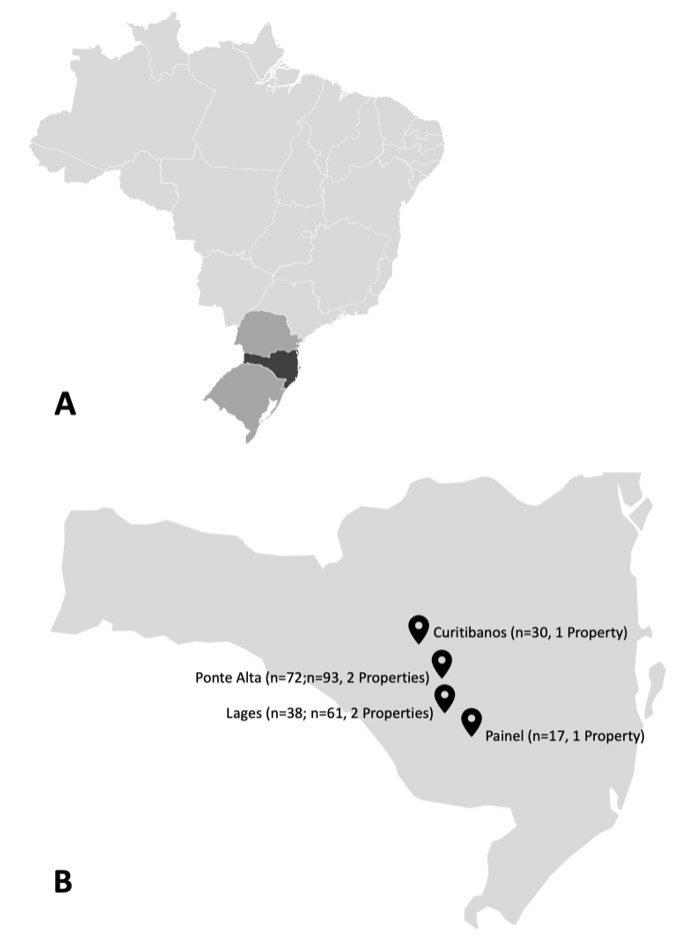
Highlighted **(A),** is Brazil’s Southern region, emphasizing the state of Santa Catarina, where the experiment took place, in the darkest shade of gray; The four municipalities where six *in situ* conservation sites for the Crioula Lageana cattle breed are located, within the state of Santa Catarina, are shown in **(B)**.

Blood samples were collected upon availability from 310 cattle of the Crioulo Lageano breed, from the 1500 animal population, with an expected prevalence of 50% at 95% confidence interval, with 5% accepted error. These animals were adult males (*n* = 32), adult females (*n* = 207), and young animals (*n* = 71; males and females), all of which were registered by the Brazilian Crioulo Lageano Cattle Breeders Association (ABCCL) (Figure 1). The animals were selected from the six *in situ* conservation nucleus properties from the following municipalities in the Plateau of Santa Catarina region: Lages (Latitude: 27°49′0″, South Longitude: 50°19′35″ West, Altitude: 930 m); Painel (Latitude: 27°55′30″ South, Longitude: 50°6′12″ West, Altitude: 1,101 m); Curitibanos (Latitude: 27°16′58″ South, Longitude: 50°35′4″ West, Altitude: 987 m); Ponte Alta (Latitude: 27°29′3″ South, Longitude: 50°22′49″ West, Altitude: 856 m) (Figure 2). The blood collection was performed by venipuncture of the external jugular vein using vacuum collection tubes with and without 10% EDTA anticoagulant to perform blood count and serum biochemistry. The samples were frozen at −20 °C until DNA extraction and PCR and IFAT were performed.

### Physical examination

Physical examination was used to verify clinical signs compatible with the clinical disease. Heart rate (HR), respiratory rate (RR), ruminal movements (RM), rectal temperature, and mucosal color were measured and evaluated accordingly to reference values stated by Dirksen et al. (1993) and Feitosa (2020).

### DNA extraction

Blood samples were subjected to DNA extraction using a commercial kit (ReliaPrep Blood gDNA Miniprep System; Promega, Madison, WI, USA), according to the manufacturer's instructions after thawing. The concentration of each DNA sample was measured using a spectrophotometer (NanoDrop 2000; Thermo Fischer, Waltham, MA, USA) after extraction, and the DNA was diluted with ultrapure water to a minimum concentration of 20 ng/μL.

### Molecular analysis

#### PCR analysis

For amplification of genomic DNA extracted from blood samples and positive controls, PCR assays were performed by amplifying a *T. vivax*-specific 210-bp SL intergenic sequence fragment in 0.2-mL microtubes containing 25 µL of a solution comprising 1 U of GoTaq Hot Start Polymerase (Promega), 8.5 pmoles of each of primers TviSL1 (5′-GCTCTCCAATCTTAACCCTA-3′ and/ TviSL2 5′-GTTCCAGGCGTGCAAACGTC-3′, (Ventura et al., 2001), 0.2 mM of nucleotides (dNTPs), 3.25 mM of magnesium chloride, 5 µL of 5X Green GoTaq Flexi buffer (Promega, Madison, USA), 3 µL of DNA (concentration between 20 and 100 ng/µL), and ultrapure water to the final volume and reagent concentration.

A negative control was used by replacing the genomic DNA with ultrapure water devoid of DNase and using the same parameters as the positive control to ensure the quality and specificity of the technique. The cycling parameters using a thermocycler (Biocycler) were an initial denaturation at 94 °C for 5 minutes; 35 cycles of 94 °C for 1 minute, 65 °C for 1 minute, and 72 °C for 3 minutes; and a final extension at 72 °C for 10 minutes. DNA extracted from *T. vivax* (GenBank: GCA_046128835.1) isolated from sheep was utilized as a positive control (Marques et al., in submission).

Electrophoresis of the amplification products (210 bp fragment) was performed in a horizontal tank using a 2% agarose gel with 1µL of Unisafe dye, and 10 µL of sample buffer (bromophenol blue and glycerol) added directly to the gel for each product. A 100 bp molecular weight marker (Ludwig) was used as a standard to determine the size of the sample bands in the initial hole of the gel. The electrical source was set to 100 volts for 40 minutes with ultraviolet light exposure to visualize the products.

#### Obtaining parasites and IFAT assay

The *T. vivax* strain utilized in this study was given by Prof. Joely from the University of Uberaba (UNIUBE) and preserved in liquid nitrogen. *Trypanosoma vivax* were thawed in a water bath at 37 °C, and a 4 mL aliquot (2.0 × 10^8^) was inoculated subcutaneously in a healthy splenectomized 8-month-old creole breed sheep that was isolated from an area with anti-insect screens inside a closed and air-conditioned shed. The animals were fed concentrate (13% protein) ad libitum. The sheep was clinically examined and found to be robust, parasite-free (Woo, 1970), and PCR-negative for trypanosomiasis. The Animal Ethics Committee of the University of Santa Catarina State authorized this project under procedure number 6926160818.

At the peak of parasitemia (12 days after parasite inoculation), 2.0 × 10^5^ parasites/mL of blood were collected in EDTA-coated vacuum tubes. Blood samples were centrifuged at 2000 × *g* for five minutes. Following centrifugation, the leukocyte layer was removed, transferred to 50-mL containers, and washed twice with physiological solution (0.9% NaCl). Five minutes were spent fixing thick smear transparencies with 30 µL of blood with acetone. The smears were wrapped in paper towels and aluminum foil before being frozen at −20 °C. The same method was used for one Creole breed sheep, as the negative control.

**Figure 3:**
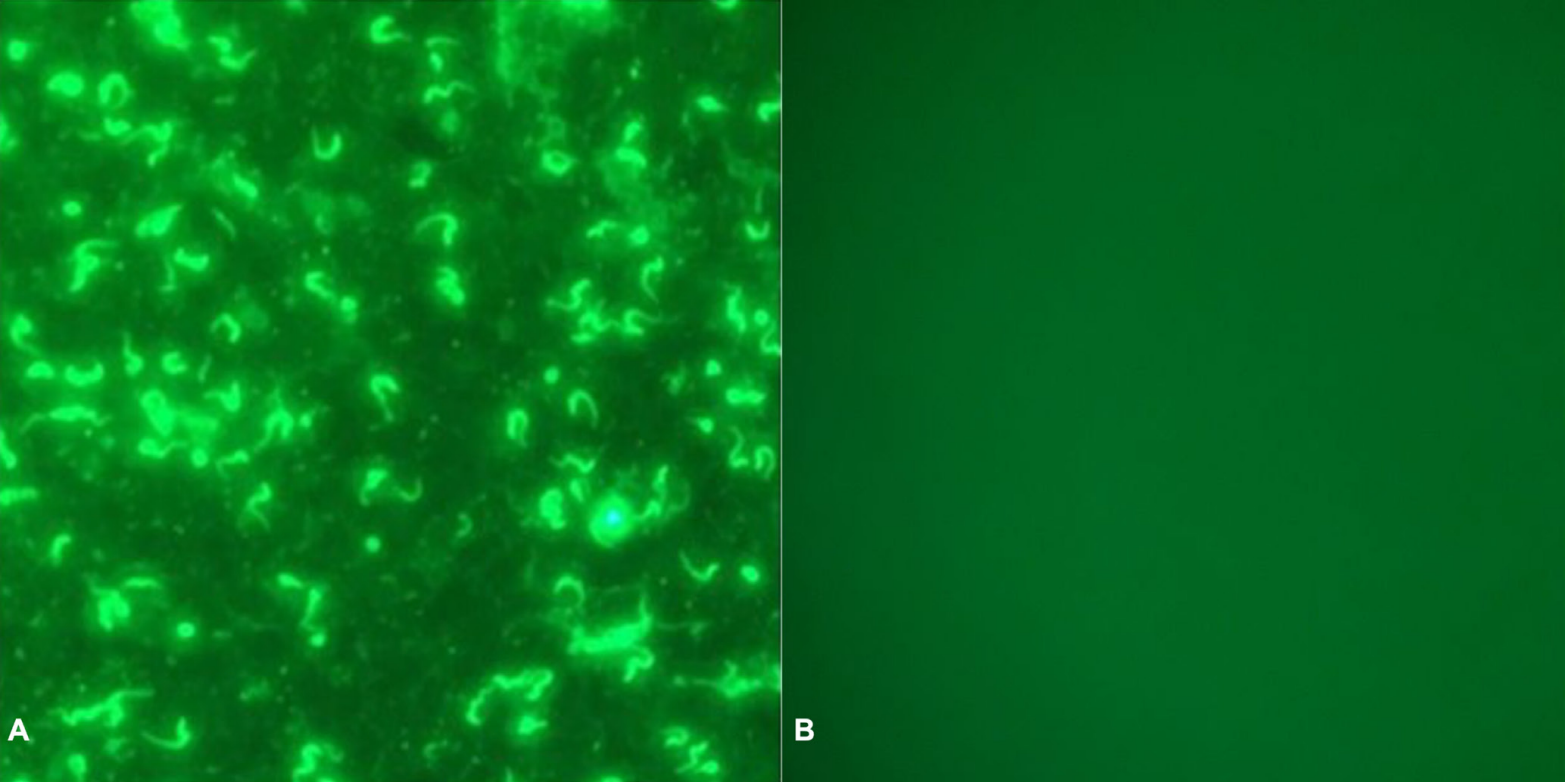
**A)** Immunofluorescence antibody test for *Trypanosoma vivax* using a positive bovine serum sample (1:80) and a FITC-labelled anti-bovine conjugate was added at 1:300 (Nikon® epifluorescent microscope). **B)** Negative control sample.

Immunofluorescence Antibody Test (IFAT) to detect antibodies was conducted according to the method described by Cuglovici et al. (2010), with a 1:80 dilution threshold, thus considering animals to be seropositive above this titration, and seronegative when below this limit. The antigen-fixed glass slidewas removed from the freezer and dried at room temperature. Positive (immune sheep serum for *T. vivax*) and negative (pre-immune serum) samples were diluted 1:80 in phosphate-buffered saline (PBS). The field samples were diluted in PBS at 1:40, 1:80, and 1:160 for screening. Diluted sera (20 µL) were placed on substrates containing the *T. vivax* antigen. The slides were incubated for 30 minutes at 37 °C in a humid chamber and washed three times with PBS. FITC-labeled anti-IgG bovine conjugate at a 1:300 dilution in PBS was added to each slide. The slides were incubated for 30 min and washed with PBS as described previously. The glass slides utilized in the immunofluorescence assay stained with DAPI (4',6'-diamino-2-phenylindole) were affixed with buffered glycerin. The analysis was performed using a Nikon epifluorescent microscope (Figure 3) (Silva et al., 2022).

### Hematology

An automated cell counter (SDH3 Labtest) was used to determine total erythrocyte and leukocyte counts, packed cell volume, hemoglobin concentration, and absolute hematimetric indices of the mean corpuscular volume (MCV), mean corpuscular hemoglobin (MCH), and mean corpuscular hemoglobin concentration (MCHC). Differential leukocyte counts were determined using microscopic analysis of blood smears stained with Romanowsky dye (Fast Panoptic). The total plasma protein (TPP) concentration was determined using refractometry (ATAGO), and the plasma fibrinogen concentration was determined using the heat precipitation method, followed by reading in a refractometer (Jain, 1993).

### Serum biochemistry

Serum biochemical profiles were analyzed based on the enzymatic activity of gamma-glutamyltransferase (GGT), aspartate aminotransferase (AST), alanine aminotransferase (ALT), alkaline phosphatase (AP), creatine phosphokinase (CK), and lactate dehydrogenase (LDH), and serum concentrations of urea, creatinine, total serum protein, albumin, globulin, cholesterol, triglycerides, and glucose. Biochemical tests were conducted using kinetic and colorimetric methods on a Labmax Plenno automatic biochemical analyzer (v2.09.05).

### Statistical analysis

The Shapiro–Wilk test was applied to all data to assess normality. The means of the clinical, hematological, and biochemical variables between seropositive and seronegative animals were compared using the Mann–Whitney test for non-parametric data. A probability error of 5% was assumed for all tests.

## Results

### Prevalence

Anti-*T. vivax* IgG antibodies found by the mere detection of titers above 1:80 in 8% (24/310) of the systemic blood samples using IFAT, with no specific titration assigned to groups of animals. Among the seropositive animals, 42% (10/24) were calves (7 females, 3 males), 37% (9/24) were cows, 17% (4/24) were heifers, and 4% (1/24) was a bull. *Trypanosoma vivax* DNA was not detected in any of the animals tested (0/310) using PCR.

### Physical examination

The variables evaluated in the clinical examination showed no difference between seropositive and seronegative animals. In both groups, the frequency of RM remained below the reference values (5–7 contractions in 5 minutes) for bovine species (Dirksen et al., 1993). The mean HRs of the two groups slightly exceeded the maximum limit of the reference value (60–80 beats per minute) for bovine species (Feitosa, 2020) but did not differ significantly among the groups (Table 1).

**Table 1:** Means, standard deviations and standard error of physical examination values of the Crioulo Lageano breed in the state of Santa Catarina, Brazil, seronegative and seropositive for *T. vivax* evaluated by the IFAT technique.

Variable	Seropositive (n=24)		Seronegative (n=286)		*p*
	Mean±SD	Std. Error	Mean±SD	Std. Error	
Temperature (°C)	39.10±0.43	0.18	38.88±0.58	0.06	0.054
RM (mov/5min)	2.82±1.80	0.65	3.55±2.23	0.35	0.171
RR (mov/min)	31.54±9.15	3.94	29.05±8.63	0.99	0.490
HR (beat/min)	89.95±22.89	5.51	86.50±24.04	2.90	0.444

* Indicates a significant difference between groups by the Mann-Whitney test.

RM: ruminal moviments; RR: respiratory rate; HR: heart rate; SD: standard deviation; Std: standard; n: number of animals; p: *p* value.

### 3.3. *Hematology*

The platelet count showed differences (*P* = 0.049) between the positive and negative groups, being higher in animals seropositive for *T. vivax*. However, it remained within the reference values for the bovine species (Jain, 1993) (Table 2).

**Table 2:** Means, standard deviations and standard error of blood count values, total plasma protein concentration, and plasma fibrinogen of Crioulo Lageano cattle in the state of Santa Catarina, Brazil, seronegative and seropositive for *T. vivax* by the IFAT technique.

Variables	Seropositive (n=24)		Seronegative (n=286)		*p*
	Mean±SD	Std. Error	Mean±SD	Std. Error	
Erythrocytes (x10^6^/µL)	7.92±1.21	0.25	7.99±1.50	0.09	0.711
Hemoglobin (g/dL)	12.43±1.86	0.37	12.81±1.78	0.10	0.322
PCV (%)	36±5.00	0.98	37.51±5.25	0.31	0.141
MCV (fL)	46.04±5.17	1.04	47.87±7.37	0.43	0.179
MCHC (%)	34.30±1.10	0.22	34.27±3.30	0.19	0.353
Platelets (x10^3^/µL)	463±236	47.47	369.70±179.75	10.62	0.049*
TPP (g/dL)	7.39±0.72	0.14	7.42±0.73	0.04	0.842
Fibrinogen (mg/dL)	422±207	40.91	383±215.22	12.79	0.312
Total leukocytes (/µL)	13440±5297	1109.69	12690±5097.09	299.82	0.718
Rods (/µL)	6±28	5.56	29.76±86	5.06	0.150
Segmented (/µL)	3080±1953	431.76	3467.56±2684	158.09	0.728
Lymphocytes (/µL)	9150±5021	1015.86	8.047.91±3411	200.89	0.512
Eosinophils (/µL)	585±559	114.22	722±711	41.95	0.483
Basophils (/µL)	4±17	3.39	10.50±42	2.46	0.517
Monocytes (/µL)	613±826	165.31	412.97±353	20.83	0.827

* Indicates a significant difference between groups by the Mann-Whitney test.

PCV: packed cell volume; MCV: mean corpuscular volume; MCHC: mean corpuscular hemoglobin concentration; TPP: total plasma protein; SD: standard deviation; Std: standard; n: number of animals; p: *p* value.

### 3.4. *Serum biochemistry*

Serum cholesterol had significantly lower values (*P* = 0.010) in animals seropositive for *T. vivax* than in seronegative ones. However, the mean values from both groups remained within the reference range (73,9–147,9 mg/dL) for the bovine species (Pogliani & Birgel, 2007) (Table 3).

**Table 3:** Means, standard deviations and standard error of serum biochemistry values of Crioulo Lageano cattle in the state of Santa Catarina, Brazil, seronegative and seropositive for *T. vivax* using the IFAT technique.

**Variable**	**Seropositive (n=24)**		**Seronegative (n=286)**		***p***
	Mean±SD	Std. Error	Mean±SD	Std. Error	
TSP (g/dL)	5.41±1.00	0.20	5.17±1.00	0.06	0.317
Globulins (g/dL)	3.09±0.86	0.17	2.86±0.88	0.05	0.242
Albumin (g/dL)	2.31±0.44	0.09	2.31±0.41	0.02	0.984
CK (U/L)	207.40±313.97	58.63	203.26±319.90	19.14	0.435
LDH (U/L)	1464.45±119.81	27.78	1440.42±178.75	10.59	0.605
Urea (mg/dL)	31.35±8.69	2.03	31.36±10.75	0.64	0.901
Creatinine(mg/dL)	1.49±0.31	0.07	1.45±0.37	0.02	0.627
AST (U/L)	58.95±12.21	2.58	60.25±21.40	1.27	0.853
AP (UI/L)	133.45±198.85	37.31	108.85±89.21	5.33	0.489
GGT (U/L)	14.05±5.83	1.19	15.97±6.97	0.41	0.235
ALT (U/L)	27.85±14.76	2.87	30.03±14.59	0.87	0.563
Cholesterol (mg/dL)	102.25±39.53	7.61	125.13±38.32	2.29	0.010*
Triglycerides (g/dL)	23.70±8.77	1.79	23.25±7.37	0.44	0.959
Glucose (mg/dL)	65.30±13.66	2.89	68.11±21.92	1.30	0.863

* Indicates a significant difference between groups by the Mann-Whitney test.

TSP: total serum protein; CK: creatine phosphokinase; LDH: lactate dehydrogenase; AST: aspartate aminotransferase; AP: alkaline phosphatase; GGT: gamma-glutamyltransferase; ALT: alanine aminotransferase; SD: standard deviation; Std: standard; n: number of animals; p: *p* value.

## Discussion

To our knowledge, this is the first study on the prevalence of *T. vivax* in Crioulo Lageano cattle. Epidemiological data characterized the population evaluated under enzootic instability (Smith et al., 2000), with 8% seroprevalence using IFAT. This means that the cattle had a certain level of exposure to the pathogen and may represent a greater susceptibility to clinical disease and the appearance of outbreaks. This was not verified in animals of this breed, as they did not present compatible clinical signs at the time of sample collection.

According to Desta et al. (2012), clinical trypanosomiasis usually appears during rainy and hot periods, favoring the growth and reproduction of vectors. The low manifestation of infection in the Crioulo Lageano breed is most likely related to this characteristic, as the predominantly cold weather in the Plateau of Santa Catarina region hinders the proliferation and reproduction of vectors (Andrade et al., 2019).

The seroprevalence was lower than that found in Santa Catarina (39%) in dairy cattle (Silva et al., 2022) and Minas Gerais (50%) (Barbieri et al., 2016) and higher than the prevalence observed in Paraíba (0%) (Costa et al., 2013). These studies were all performed using the IFAT technique, but remained within the seroprevalence found on the African continent (5–15%), an area endemic to biological and mechanical vectors (Fetene et al., 2021). These values are lower than those observed in the Amazon (53%) in buffaloes using PCR (Garcia Pérez et al., 2020).

The variation in prevalence between the aforementioned studies may be related to different forms of management, including the use of antiparasitic drugs, as well as factors related to the vectors, such as their distribution and vectorial competence (Garcia Pérez et al., 2020). Importantly, these studies were conducted during different seasons of the year, implying varied infection rates as the population of vectors increased according to the climate season (Onditi et al., 2007).

Another key factor to be considered is the great variation in the breeds used in these studies due to the phenomenon called trypanotolerance, which may be present in animals of the Crioulo Lageano breed, indicating that exposure to the protozoa does not necessarily cause clinical disease. This trait may benefit sustainable cattle farming in endemic areas, making this breed a valuable genetic resource (D’Ieteren et al., 1998).

The differences between the methodologies need to be verified because IFAT, the only test used in these studies, evaluates the presence of antibodies against the agent to determine an animal’s seropositivity (Guerra et al., 2013), whereas PCR enables the detection of the presence of the agent itself parasitizing the blood of the animals at the time of analysis, indicating an active infection (Fidelis et al., 2019).

A higher prevalence has been reported using serological techniques such as IFAT than using molecular techniques such as PCR. This is due to the persistence of antibodies for several months, even after treatment (Fetene et al., 2021). These values differ from those obtained in the present study, resulting in an 8% prevalence using the IFAT technique.

Although there were seropositive animals, neither diagnostic method detected animals infected with *T. vivax*. Serological tests must be associated with molecular tests, and active infections can be identified through detecting the agent’s DNA using PCR and antibodies originating from infections that occur at some point in the host’s life, with no active infection or even chronic infections presenting with low parasitemia using IFAT (Fidelis et al., 2019). This study conducted PCR reactions following the methodology of Ventura et al. (2001), which exhibited a sensitivity of 500 fg of DNA, in contrast to LAMP techniques that have a detection limit of 1 pg of DNA, equivalent to one trypanosome per mL of blood (Njiru et al., 2011).

Nevertheless, all animals were considered clinically healthy, without clinical signs compatible with trypanosomiasis, as demonstrated by Silva et al. (2022), who observed that positive animals remained asymptomatic carriers. There was a decrease in RM and a slight increase in the mean HR in both the seropositive and seronegative groups compared to the reference values. These were not regarded as relevant, given the weak correlation between clinical signs and infection (Silva et al., 2022).

Animals clinically ill with trypanosomiasis caused by *T. vivax* show non-specific signs, such as fever, anemia, inappetence, weight loss, progressive weakness, apathy, miscarriage, hemorrhagic syndromes, and neurological alterations, such as motor incoordination, muscle tremors, and blindness (Andrade et al., 2019). These can also be observed in symptomatic animals infected with other hemoparasites, such as *Babesia* spp. and *Anaplasma* spp., hindering the ability to make accurate field diagnoses (Barbieri et al., 2016).

The absence of clinical disease in the Crioulo Lageano herds does not eliminate the need to compare clinical variables between the seropositive and seronegative groups using IFAT. There was no statistically significant difference (*P* < 0.05) among the groups (Table 1). Hence, the probable previous natural exposure to *T. vivax* did not affect these variables and led to the development of antibodies in some animals.

The occurrence of disease outbreaks or experimental infections would enable for a more accurate comparison of clinical, hematological, and biochemical variables between healthy and sick animals, making it possible to observe the different manifestations between animals that develop the clinical form of the disease and those that become subclinically infected.

In agreement with Silva et al. (2022), *T. vivax* seropositivity was not associated with anemia in the animals, with no difference between seropositive and seronegative groups, showing that previous exposure to the agent does not cause changes as expected when the clinical disease manifests. Animals infected with *T. vivax* present with anemia due to intra- and extravascular hemolysis, decreased or inhibited erythropoiesis, and hemorrhage (Frange, 2013). This hematological abnormality is most commonly associated with trypanosomiasis caused by *T. vivax* (Batista et al., 2008). Maxie et al. (1979) demonstrated the development of hypochromic macrocytic anemia associated with thrombocytopenia in bovines subjected to experimental infection.

The number of platelets was higher in the seropositive group, but the values of both groups were within the reference values for the species. No explanation is available for this variation in the consulted literature, as these values were not correlated with the infection (Jain, 1993). Cattle with trypanosomiasis caused by *T. vivax* commonly have thrombocytopenia related to the consumption of platelets for the formation of microthrombi induced by the parasite (Frange, 2013).

Among serum biochemical variables, cholesterol levels were lower in animals seropositive for *T. vivax*. Despite differences between the groups, the mean cholesterol values of the positive and negative animals remained within the reference values for the species (Pogliani & Birgel, 2007). According to Kaneko (1997), the cholesterol level of the negative group remained slightly above the reference values; however, this did not correlate with any form of clinically visible disorder. Lower cholesterol values in positive animals can indicate that their energetic metabolism is directed towards the oxidative processing of lipids, which may occur due to inadequate energy supply in the diet or consumption of energy reserves by trypanosomes in subclinical cases (Kadima et al., 2000).

The differences found upon testing are considered non-specific, and no concrete explanation for such alterations has been found in the literature. Therefore, these differences were not solely associated with infection or seropositivity to the *T. vivax* agent. Some cattle breeds are relatively resistant to trypanosomiasis because of their natural resistance to the infection and their ability to remain as subclinical carriers, which are clinically healthy even in the presence of the agent, a phenomenon called trypanotolerance (D’Ieteren et al., 1998; Frange, 2013).

The breed effect is closely related to this condition; the Sheko breed (taurine origin) in Africa is considered trypanotolerant (Desta et al., 2012; Schenk et al., 2001), as is the N’Dama breed (D’Ieteren et al., 1998). The inhospitable environmental, climatic, and nutritional conditions of Crioulo Lageano cattle can be a determining factor in the characteristic of being possibly trypanotolerant, as this particularity has a genetic and environmental origin and may vary as a function of stress conditions and nutritional status (Garcia Pérez et al., 2020). This characteristic has been observed in other locally adapted cattle breeds from South America, such as the Curraleiro-pé-duro (Mendonça et al., 2024).

Resistance or tolerance to diseases is among the most valuable characteristics of locally adapted breeds and is a powerful control and prevention tool when used in selection and genetic breeding programs, especially in regions where the disease is endemic (Soares Fioravanti et al., 2020). The Crioulo Lageano breed falls within this category, as it becomes very productive and adapted, given how it undergoes natural selection under adverse conditions (Mariante & Cavalcante, 2000).

The results of the clinical, hematological, and serum biochemical evaluations of animals seropositive for *T. vivax* suggested that previous exposure did not trigger the clinical disease or persistent infection, strengthening the proposition that the Crioulo Lageano breed may also have the resistance observed in African breeds, also originating from *Bos taurus* animals (D’Ieteren et al., 1998), and in other South American breeds, with the same origins (Mendonça et al., 2024).

Another point to consider is the possibility that the results obtained through conventional PCR are underestimated, given how a more targeted approach, as prior GADPH testing, would enable the assessment of the presence of PCR inhibitors in the exctracted DNA that may impede the parasite´s detection (Castilho et al., 2021). LAMP has been demonstrated to have a greater detection capability in detecting *T. vivax* in cattle, mainly in aparasitemic phase (Cadioli et al., 2015). Its association with ELISA may increase the reliability of this screening (Castilho et al., 2021), as *T. vivax* has been shown to take refuge in skin and adipose tissues, lowering their concentration on the bloodstream (Machado et al., 2021). Moura et al. (2024) observed the same in naturally infected buffaloes and cattle in the Brazilian Amazon.

Further studies may determine whether the possible resistance or tolerance to *T. vivax* by animals of the Crioulo Lageano breed is associated with the breed in particular, or simply with bovine lineages, or even environmental, climatic, and nutritional factors. The presence of genes that confer this characteristic to animals (Duangjinda et al., 2013) would help to determine whether resistance exists in this breed group.

Antigenic variations of the parasite are also a factor possibly capable of interfering with the manifestations of cattle in the face of the infection (Frange, 2013). Thus, detecting these variations would help prove the supposed resistance or tolerance of animals of the Crioulo Lageano breed to diseases in general (Casa et al., 2020). This raises the possibility and importance of further studies regarding investigating the possible tolerance or resistance of Crioulo Lageano breed cattle to infection by *T. vivax* since no significant alteration was reported in this study in animals seropositive for the agent.

## Conclusions

The seroprevalence of *T. vivax* determined using IFAT was 8%, which is characteristic of enzootic instability in Crioulo Lageano cattle in the state of Santa Catarina. No animals tested positive for *T. vivax* using PCR. The cattle were considered asymptomatic, with few clinical alterations, and the test results confirmed previous exposure to the agent. The absence of clinical disease, along with the presence of seropositive animals, can lead to further studies and the possible characterization of Crioulo Lageana cattle as a trypanotolerant breed.
